# Treatment of Cutaneous Leishmaniasis in Persian Medicine

**Published:** 2017-10

**Authors:** Omid MOZAFARI, Seyed Afshin SHOROFI, Mohammad Reza SHIRZADI, Seyde Sedighe YOUSEFI

**Affiliations:** 1. Traditional and Complementary Medicine Research Center, Student Research Committee, Mazandaran University of Medical Sciences, Sari, Iran; 2. Dept. of Traditional Persian Medicine, School of Medicine, Mazandaran University of Medical Sciences, Sari, Iran; 3. Traditional and Complementary Medicine Research Center, Mazandaran University of Medical Sciences, Sari, Iran; 4. Adjunct Research Fellow, Flinders University, Adelaide, Australia; 5. Center for Communicable Diseases Control (CDC), Ministry of Health and Medical Education, Tehran, Iran

## Dear Editor-in-Chief

Cutaneous leishmaniasis (CL) is considered an important public health problem in many countries. However, there are numerous drugs available for the treatment of CL, at present, first-line drugs including pentavalent antimonials exhibit for problems such as prolonged systemic therapy, high toxicity and less affectivity against various forms of the disease in patients. Moreover, second-line drugs also have limitations in their use because of the high cost, prolonged length of therapy and adverse reactions. For these reasons, development of new drugs or combination therapy for the treatment of CL is necessary (1). Persian Medicine (PM) can be used in conjunction with conventional medicine for treatment of the disease (2). PM consists of total of all the knowledge and practices used in diagnosis, prevention and elimination of diseases in Persia from ancient times to the present, based entirely on practical experience and observations passed down from generation to generation (3). According to PM, each one has a unique characteristic named their temperament or Mizaj, determined by physiological, morphological and psychological features. Everybody is considered in a healthy state when Mizaj has been balanced. When diseases occur, the Mizaj becomes imbalanced. From this viewpoint a package of certain measures are recommended for the treatment of diseases. –For CL, these treatments can be categorized in two categories, general measures and topical treatments:

1. General measures are used to reverse the Mizaj of patient to a cooler and wetter status by applying the following recommendations:
  - Consumption of wet and cold drinks such as sour pomegranate, apple or rhubarb juice (4–6).
  - Consumption of wet and cold foods such as Kashkab (6) and avoidance of very spicy, salty or sweet foods and beef and salted meat (4, 7).
  - Reduction of the temperature of surrounding environment, advising patients in the past to switch from areas with hot weather to those with cooler weather (4–6).
Topical treatments:
  - Draining lesion and removing dead tissue until healthy tissue is revealed by; Washing the wound with vinegar or a combination of vinegar and Gele-armani, so that the lesion dries and dead tissue can be separated layer by layer. Stronger compounds may be recommended for this purpose if needed.
    Even use of hot instruments on lesion and burning the wound followed by rubbing the wound may be used in order to achieve healthy tissue, with due regard to the lack of damage to other adjacent tissues.
  - When healthy tissue is revealed, wound healing drugs (Modamel drugs) must be used to repair the skin (4–8).

Modamel drugs, such as a combination of Aloe vera, Tragacanth and Dracaena cinnabari are a group of drugs that can repair wounds (8). Persian Medicine could offer different techniques for the treatment of CL and pay closer attention to this viewpoint with evaluation of the effectiveness of the drugs and methods through clinical trial studies.
